# Exploring the roles of ncRNAs in prostate cancer via the PI3K/AKT/mTOR signaling pathway

**DOI:** 10.3389/fimmu.2025.1525741

**Published:** 2025-03-18

**Authors:** Rongwang Guo, Liji Shi, Yonghui Chen, Canling Lin, Weihua Yin

**Affiliations:** ^1^ Nanchang University, 999 University Avenue, Honggutan District, Nanchang, China; ^2^ School of Chemical and Biological Engineering, Yichun College, Yichun, China; ^3^ Department of Oncology, Baoan Central Hospital of Shenzhen, Bao’ an District, Shenzhen, China

**Keywords:** prostate cancer, ncRNA, PI3K/AKT/mTOR, metastasis, tumor growth

## Abstract

Although various treatment options are available for prostate cancer (PCa), including androgen deprivation therapy (ADT) and chemotherapy, these approaches have not achieved the desired results clinically, especially in the treatment of advanced chemotherapy-resistant PCa. The PI3K/AKT/mTOR (PAM) signaling pathway is a classical pathway that is aberrantly activated in cancer cells and promotes the tumorigenesis, metastasis, resistance to castration therapy, chemoresistance, and recurrence of PCa. Noncoding RNAs (ncRNAs) are a class of RNAs that do not encode proteins. However, some ncRNAs have recently been shown to be differentially expressed in tumor tissues compared with noncancerous tissues and play important roles at the transcription and posttranscription levels. Among the types of ncRNAs, long noncoding RNAs (lncRNAs), microRNAs (miRNAs), circular RNAs (circRNAs), and Piwi-interacting RNAs (piRNAs) can participate in the PAM pathway to regulate PCa growth, metastasis, angiogenesis, and tumor stemness. Therefore, ncRNA therapy that targets the PAM signaling pathway is expected to be a novel and effective approach for treating PCa. In this paper, we summarize the types of ncRNAs that are associated with the PAM pathway in PCa cells as well as the functions and clinical roles of these ncRNAs in PCa. We hope to provide novel and effective strategies for the clinical diagnosis and treatment of PCa.

## Graphical Abstract

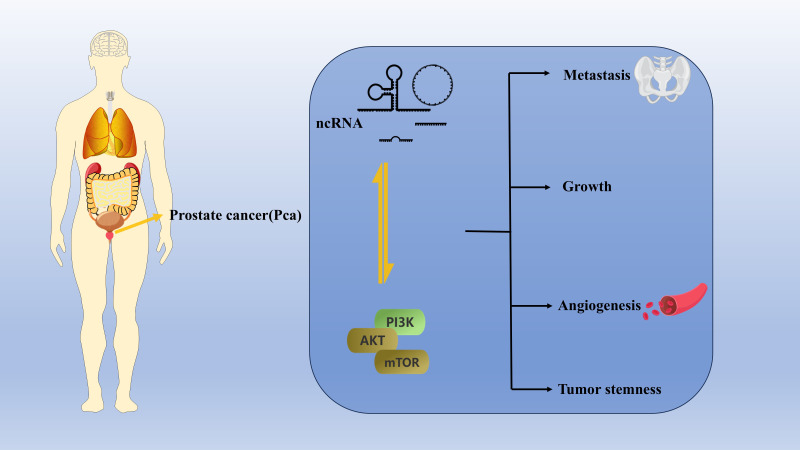

## 1 Introduction

According to Global Cancer Statistics 2022, prostate cancer (PCa) is the fourth most common malignant tumor worldwide (incidence of 7.3%), and it is the second most prevalent malignant tumor in men (prevalence of 14.2%) and the fifth leading cause of malignant tumor-related death in men (7.3% of tumor-related deaths) (1). Moreover, PCa has a heterogeneous regional distribution, and it is the most common cancer in 118 countries (e.g., around northern Europe, Australia-New Zealand, and the Caribbean) (1). Advanced PCa is the main cause of PCa-related death, and it is treated mainly with androgen deprivation therapy (ADT) and chemotherapy; however, advanced PCa is susceptible to drug resistance development after a period of treatment (2). Therefore, understanding the mechanisms underlying PCa development and progression is important for preventing and treating this condition.

The PI3K/AKT/mTOR (PAM) signaling pathway is highly conserved in eukaryotes, and it is the pathway that is most often abnormally activated in malignant tumors (3). Studies have shown that aberrant activation of the PAM pathway may be the result of gene mutation/amplification, epigenetic modification, and aberrant regulation of other signaling pathways; recently, the regulation of epigenetic modifications, which mainly include DNA methylation, histone posttranslational modification, and ncRNAs regulation, has become a popular research topic (3). The PAM signaling pathway regulates tumor cell proliferation, apoptosis, metastasis, and angiogenesis; thus, the PAM signaling pathway has become a target for the treatment of malignant tumors (4). For example, XL147 (an inhibitor of PI3K class 1 molecules) can inhibit the proliferation of breast cancer and PCa cells; inhibit the migration, invasion, and angiogenesis of melanoma cells *in vitro*; and increase the efficacy of chemotherapy in mice with breast cancer (5). Inhibition of the PAM signaling pathway enhances the immune checkpoint blockade response. Understanding the potential mechanisms underlying the aberrant activation of the PAM signaling pathway in PCa cells could provide valuable insights into overcoming PCa.

Noncoding RNAs (ncRNAs) are a class of RNAs that are not translated into proteins; these molecules are involved in gene modification, RNA transcription, protein translation, and functional travel (6). Current studies have briefly described the roles of several categories of ncRNAs, such as long noncoding RNAs (lncRNAs), microRNAs (miRNAs), circular RNAs (circRNAs), and piwi-interacting RNAs (piRNAs), in PCa. These ncRNAs show promise as diagnostic and prognostic markers of PCa (7). For example, the lncRNA *SChLAP1* is highly expressed specifically in PCa cells and is enriched mainly in high-risk patients and metastatic patients; thus, *SChLAP1* can be used to predict and diagnose the occurrence and metastasis of PCa (8). In addition, miR-145 overexpression can improve the radiosensitivity of PCa cells by impairing DNA gene repair (9), suggesting that the modulation of ncRNA expression is a promising strategy for treating PCa. In recent years, many studies have focused on the mechanism underlying ncRNA functions in PCa, and it has been shown that ncRNAs can regulate the growth, apoptosis, epithelial−mesenchymal transition (EMT), metastasis, immune microenvironment, and other behaviors of cancer cells by regulating the PAM signaling pathway (10); therefore, ncRNAs that are related to PAM signaling are likely to constitute a large class of targets for treating PCa.

In conclusion, in this review, we elucidate in detail the complex relationship between ncRNAs and the PAM signaling pathway in the development of PCa. Notably, to date, no relevant review has comprehensively reported the ncRNAs that are involved in the PAM signaling pathway in PCa cells.

## 2 Types of ncRNAs that are linked to the PAM signaling pathway

In prostate cancer, ncRNAs associated with the PAM signaling pathway can be divided into four categories by class: lncRNAs, miRNAs, circRNAs, and piRNAs (**Table 1**).

**Table 1:** Type and role of ncRNAs associated with the PAM signaling pathway in PCa progression.

	ncRNA	Effect on the PAM pathway	Function	Signaling network	Ref.
LncRNA	LINC01088	Activate	Promote cell viability, migration, and invasion	miR-22/CDC6	(11)
	RHPN1-AS1	Activate	Promote cell proliferation and invasion Inhibit apoptosis and autophagy	miR-7-5p/EGFR	(12)
	DANCR	Activate	Promote cell proliferation, G1-S transformation, EMT, migration, and invasion	miR-185-5p/LASP1	(13)
	MBNL1-AS1	Inhibit	Inhibit cell proliferation, migration, and invasion	miR-181a-5p/PTEN	(14)
	ZEB1-AS1	Activate	Promote cell viability, migration, and invasion	miR-342-3p/CUL4B	(15)
	HCG11	Inhibit	Inhibit cell viability, migration, invasion, EMT, and tumor growth in nude mice Promote apoptosis	miR-543	(16)
	GAS5	—	Inhibit cell viability and proliferation Promote cell autophagy	miR-21/PDCD4, PTEN miR-1284/AKT	(17)
	SNHG3	Activate	Promote cell proliferation, migration, invasion, EMT, and tumor growth in nude mice	miR-1827	(18)
	SNHG1	Activate	Promote cell viability, proliferation, migration, invasion, and tumor growth in nude mice Inhibit apoptosis and autophagy	miR-377-3p/AKT2, EZH2	(19, 20)
	CASC11	Activate	Promote cell proliferation, migration, and invasion	miR-145/IGF1R	(21)
	CHRF	Activate	Promote cell proliferation and EMT Inhibit apoptosis	miR-10b	(22)
	LINC00963	Activate	Promote cell viability, migration, and invasion Inhibit apoptosis	EGFR	(23)
	PCAT1	Activate	Promote cell viability, proliferation, and tumor growth in nude mice Inhibit apoptosis	FKPB51–IKKα–PHLPP	(24)
	NEAT1	Activate	Promote cell viability	SRC3/IGF1R	(25)
	MALAT1	Activate	Promote cell viability, proliferation, migration, invasion, EMT, and tumor growth in nude mice Inhibit apoptosis	—	(26, 27)
	LINC00460	Activate	Promote cell proliferation, and cell cycle progression Inhibit apoptosis	—	(28)
	SNHG25	Activate	Promote cell proliferation, migration, invasion, and tumor growth in nude mice Inhibit apoptosis	—	(29)
	lncRNA-ATB	Activate	Promote cell proliferation and EMT	—	(30)
	PlncRNA-1	Activate	Promote cell proliferation, migration, invasion, and cell cycle Inhibit apoptosis	PTEN	(31)
	LINC01296	Activate	Promote cell proliferation, migration, invasion, and EMT	—	(32)
	AC245100.4	Activate	Promote cell proliferation	PAR2	(33)
	MIR4435-2HG	Activate	Promote cell proliferation, migration, invasion, and tumor growth in nude mice	ST8SIA1/FAK	(34)
	UCA1	Activate	Promote cell proliferation and cell cycle	—	(35)
	SPRY4-IT1	Activate	Promotes cell proliferation and cell cycle Increase cellular resistance to hypoxia	—	(36)
	GDPD4-2	Inhibit	Inhibit tumor growth in nude mice	—	(37)
miRNA	miR-139-5p	Inhibit	Inhibit cell viability and EMT	ZNF217	(38)
	miR-151	Inhibit	Inhibit cell viability, migration, and invasion Promote apoptosis	—	(39)
	miR-101-3p	Inhibit	Inhibit cell viability, migration, and invasion Promote apoptosis	CUL4B	(40)
	miR-129	Inhibit	Inhibit cell viability, proliferation, EMT, migration, invasion, and tumor growth in nude mice Promote apoptosis	ETS1	(41)
	miR-101	Inhibit	Inhibit cell viability Promote apoptosis	RLIP76	(42)
	miR-4638-5p	Inhibit	Inhibit cell proliferation, tumor angiogenesis, and tumor growth in nude mice Promote apoptosis	Kidins220	(43)
	miR-133a-3p	Inhibit	Inhibit tumor stemness Attenuate resistance to anoikis	EGFR, FGFR1, IGF1R, MET	(44)
	miR -7	Inhibit	Inhibit tumor stemness, cell proliferation, tumor growth in nude mice, and cell cycle progression	KLF4	(45)
	miR-218	Inhibit	Inhibit tumor angiogenesis, tumor growth in nude mice, cell viability, proliferation, migration, invasion, EMT, and cell cycle progression	RICTOR, GAB2, LGR4	(46–48)
	miR-4534	Activate	Promote cell proliferation, migration, invasion, cell cycle, and tumor growth in nude mice Inhibit apoptosis	PTEN	(49)
	miR-21	Activate	Inhibit apoptosis in chemoresistant cells	PTEN	(50)
	miR-182	Activate	Promote cell proliferation, tumor growth in nude mice	ST6GALNAC5	(51)
	miR -149	Inhibit	Inhibit cell proliferation, migration, invasion, EMT, and cell cycle Promote apoptosis	AKT1	(52)
	miR-125b	Inhibit	Inhibit cell migration and invasion	ErbB2/3	(53)
	miR-22	Activate	Promote cell proliferation, migration, and invasion	PTEN	(53)
	miR-19b	—	Promotes cell proliferation and cell cycle	PTEN	(54)
	miR-23b	—	Promotes cell proliferation and cell cycle	PTEN	(54)
	miR-26a	—	Promotes cell proliferation and cell cycle	PTEN	(54)
	miR-92a	—	Promote cell proliferation, migration, invasion, and cell cycle Inhibit apoptosis	PTEN	(54, 55)
	miR-151a-3p	Inhibit	Inhibit cell proliferation and migration	NEK2	(56)
	miR-188-5p	Inhibit	Inhibit cell proliferation, migration, invasion, tumor growth and metastasis in nude mice	LAPTM4B	(57)
	miR-605	Inhibit	Inhibit cell proliferation and cell cycle	EN2/PTEN	(58)
	miR-137	—	Inhibit H3K4 methylation	KDM5B	(59)
	miR-106a-5p	—	Promote cell migration and invasion	TIMP2	(60)
	miR-106b	—	Promote cell growth (in the presence of prolactin)	P21	(61)
	miR-27a	—	Inhibit cell proliferation, cell cycle, and migration Promote apoptosis	MAP2K4	(62)
	miR-135a	—	Inhibit cell proliferation, cell cycle, and migration Promote apoptosis	RBAK, MMP11	(63)
circRNA	CircSMARCC1	Activate	Promote cell proliferation, migration, invasion, tumor growth and metastasis in nude mice	miR-1322/CCL20	(64)
	circMBOAT2	Activate	Promote cell proliferation, migration, invasion, tumor growth and metastasis in nude mice	miR- 1271-5p/mTOR	(65)
	circ-ITCH	Inhibit	Inhibit cell proliferation, migration, and invasion	miR-17	(66)
piRNA	piR-001773	Activate	Promote cell proliferation, migration, invasion, and tumor growth in nude mice	PCDH9	(67)
	piR-017184	Activate	Promotes cell proliferation, migration, invasion, and tumor growth in nude mice	PCDH9	(67)

### 2.1 LncRNAs

LncRNAs are a class of RNAs that are more than 200 nucleotides in length and do not encode proteins; most of the lncRNAs that are involved in the PAM signaling pathway in PCa cells, such as *LINC01088 (* 11), *MALAT1 (* 26, 27), *RHPN1-AS1 (* 12), *DANCR (* 13), *ZEB1-AS1 (* 15), and *LINC004600 (* 28), are oncogenes that can promote cancer cell viability, proliferation, metastasis, etc. In PCa cells, the lncRNAs that participate in the PAM signaling pathway as antioncogenes mainly include *MBNL1-AS1*, *HCG11*, and *GDPD4-2*, all of which are underexpressed in PCa cells compared with normal prostate cells (14, 16, 37). The lncRNAs that are the focus of this project regulate the PAM signaling pathway at multiple levels, and the direct targets that have been well studied include miRNAs, coding RNAs, and proteins. The current study indicates that most lncRNAs act as sponges for miRNAs, decreasing their expression. Nevertheless, some lncRNAs, such as the lncRNA *CHRF*, which positively regulates miR-10b expression, have also been shown to increase the transcription of miRNAs (22). Second, lncRNAs can also affect mRNAs; for example, *LINC00963* can transactivate Epidermal growth factor receptor (EGFR) (23). In addition, lncRNAs can interact with proteins that are involved in the PAM pathway; for example, the lncRNA *SNHG* interacts with EZH2 (19), the lncRNA *PCAT1* competes with PHLPP for binding to FKPB51 (31), and the lncRNA *NEAT1* binds to SRC3 at the promoter of IGF1R (25).

### 2.2 MiRNAs

MiRNAs can be fully or incompletely complementary to mRNAs, and their interaction with mRNA targets can lead to the failure or attenuation of mRNA translation. The target mRNAs of most of the miRNAs that regulate the PAM signaling pathway in PCa cells have been identified, and the most frequently targeted mRNA is Phosphatase and tensin homolog(PTEN) (14, 17, 49, 53–55). The miRNAs that regulate the PAM signaling pathway in PCa cells can be classified as oncogenes and antioncogenes according to their functions, and most of them are antioncogenes. Other genes, such as *miR-185-5p*, *miR-92a*, *miR-4534, miR-21, miR-22, miR-26a, miR-543, miR-10b*, and *miR-182*, are oncogenes, and the first six of these molecules can target PTEN (14, 16, 22, 49–51, 53–55). Five miRNAs were also found to be regulated by the PAM signaling pathway, which will be described in 2.3 later.

### 2.3 CircRNAs

The main function of circRNAs, which are ring-shaped ncRNAs, is to interact with ncRNAs or proteins to participate in regulating transcription and posttranscription processes, and a few circRNAs have recently been shown to serve as templates for translation (68). Current studies have shown that in PCa cells, circRNAs regulate the PAM signaling pathway mainly by acting as miRNA sponges; for example, *cir-ITCH* and *miR-17* inhibit each other to negatively regulate the PAM pathway (66). Moreover, *circMBOAT2* and *circSMARCC1* positively regulate the PAM signaling pathway via *miR-1271-5p* and *miR-1322*, respectively (64, 65). circRNAs are mainly stable in the cytoplasm in the form of covalent monocycles and are expressed in tissue- and cell-specific patterns; thus, they can be used as diagnostic markers (68). For example, *circMBOAT2* and *circSMARCC1* are highly expressed in PCa cells, and higher plasma levels of *CircSMARCC1* have been detected in PCa patients than in noncancer patients (64, 65). In addition, circRNAs are involved in the regulation of the immune microenvironment; *circSMARCC1* increases the number of M2 macrophages in PCa tissues by recruiting M2 macrophages via the CCL20−CCR6 axis and promoting M2 macrophage polarization (64). M1/M2 macrophages can be viewed as a scale in tumors, with M1 macrophages playing an antitumor role. M2 macrophages promote tumor development by regulating angiogenesis and lymphangiogenesis, immunosuppression, hypoxia induction, and cancer cell proliferation and metastasis (69). Therefore, circRNA imbalance is one of the etiological factors of PCa at the molecular level, and because of the specific expression and stable presence of circRNAs, it will be possible to identify circRNAs that can be used as high-quality diagnostic markers.

### 2.4 PiRNAs

piRNAs are a class of small noncoding RNAs that are 24–31 nucleotides in length. piRNAs form PIWI complexes with piwi proteins that are involved in transcriptional and posttranscriptional regulation (70). PCDH9 can competitively inhibit the formation of PI3K by binding to P85α, which is a regulatory subunit of PI3K, thereby affecting the phosphorylation of PIPs (67). In PCa cells, *piR-001773* and *piR-017184* can bind to PIWIL4 to form a PIWI/piRNA complex and silence the expression of PCDH9, thereby activating the PAM signaling pathway to promote PCa development (67). In addition, *piR-001773* and *piR-017184* were found to be correlated with the T stage and Gleason score of PCa patients, and high expression of *piR-001773* and *piR-017184* suggest a poor prognosis (67).

## 3 Functions of ncRNAs in the PAM signaling pathway in PCa cells

### 3.1 Metastasis

Metastatic PCa strongly affects the prognosis of PCa patients and is the leading cause of death in advanced PCa patients. Cancer cell metastasis occurs through a certain process; this process starts with cancer cell acquisition of a migratory and invasive phenotype through EMT, after which the cancer cell passes through the extracellular matrix (ECM) and stromal cells to dislodge them from their primary foci (71). Upon loss of epithelial cell contact with the ECM, cancer cells undergo a form of cysteine-dependent apoptosis that is induced by death receptors and mitochondria that is known as anoikis (72). However, the resistance of cancer cells to anoikis leads to the progression of tumor metastasis (73). Therefore, EMT, anoikis, and migration and invasion assays are commonly used in experiments to assess the metastatic ability of tumors.

#### 3.1.1 Inhibition of metastasis

**Figure 1:**
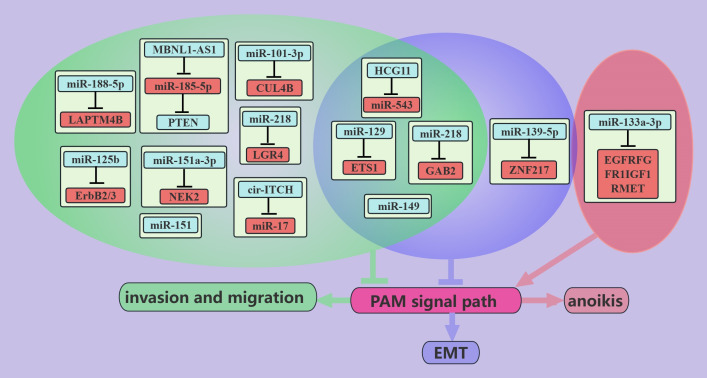
ncRNAs inhibit PCa metastasis through the PAM signaling pathway. This figure illustrates that ncRNAs associated with the PAM signaling pathway inhibit PCa metastasis by regulating invasion, migration, anoikis, and EMT. Blue represents the role of suppressing the PAM signaling pathway. Red represents roles that facilitate the PAM signaling pathway. Cyan color represents an association with invasion and migration, purple color represents an association with EMT, and light red color represents an association with anoikis.

ncRNAs associated with the PAM signaling pathway inhibit PCa metastasis (**Figure 1**). The skeletal system is the most common site of the distant metastasis of PCa. *miR-133a-3p* was shown to be expressed at lower levels in PCa bone metastatic tissues than in nonmetastatic cancer tissues, and its expression is negatively correlated with bone metastasis-free survival in PCa patients (44). Further studies revealed that overexpression of *miR-133a-3p* attenuates anoikis resistance and thus inhibits PCa bone metastasis, which may be mediated by *miR-133a-3p* targeting EGFR, FGFR1, IGF1R and MET to regulate the PAM signaling pathway (44). These findings suggest that *miR-133a-3p* may be a potential target for the treatment of PCa bone metastases. PTEN, which is a classic inhibitor of the PAM signaling pathway, is lacking in up to 60% of PCa tumors (74). The *MBNL1-AS1* inhibits the invasion and migration of PCa cells through the *miR-181a-5p*/PTEN axis [18]. Cullin 4B (CUL4B) is a scaffold for the Cullin4B-Ring E3 ligase complex (CRL4B), which is involved in protein hydrolysis (75). CUL4B is highly expressed in PCa patients, and *miR-101-3p* can target CUL4B to inhibit its expression and thus inhibit PCa migration and invasion; this is mediated by inhibiting the PAM signaling pathway (40). Yongguang Jiang reported that *miRNA-149* directly inhibits AKT1 mRNA expression in CRPC, thus inhibiting tumor EMT, migration, and metastasis; however, this process does not involve PI3K (52).

The expression level of *miR-218* in metastatic PCa tissues is much lower than that in primary PCa tissues (46), and *miR-218* can inhibit the EMT, migration, and metastasis of cancer cells via GAB2/PI3K/AKT/GSK-3β (47). Recent studies have shown that PCa is associated with chronic inflammation (76), and leucine-rich repeat-containing G protein-coupled receptor (LGR) 4 is a key molecule in the progression of PCa whose expression is induced by the proinflammatory factor IL-6 [69]. *miR-218* inhibits the migration and metastasis of IL-6-treated PCa cells by directly targeting LGR4 to inhibit the PAM pathway (48). ETS1, which is an independent prognostic molecule in breast cancer (77), is a proto-oncogene that is expressed mainly in triple-negative breast cancers (78). Higher expression of ETS1 was observed in PC cells than in noncancerous cells, especially in desmoplasia-resistant prostate cancer cells (41). miR-129 can directly target ETS1 and thus negatively regulate the PAM signaling pathway, thereby inhibiting the EMT, migration, and invasion of PCa cells (41).

In addition, other ncRNAs that are associated with the PAM signaling pathway, such as *HCG11*/*miR-543 *(16), *miR-125b *(53), *miR-151a-3p *(56), *miR-188-5p *(57), *miR-151 *(39), and *miR-139-5p *(38), inhibit the migration and metastasis of PCa cells through different pathways. *cir-ITCH* was found to negatively regulate the PAM signaling pathway and thus inhibit the EMT, migration, and invasion of PCa cells, possibly through *miR-17 *(66). In addition, some miRNAs are negatively regulated by the PAM signaling pathway, such as *miR-27a* and *miR-135a*, which inhibit migration and invasion of PCa cells through MAP2K4 and MMP11, respectively (62, 63).

#### 3.1.2 Promotion of metastasis

**Figure 2:**
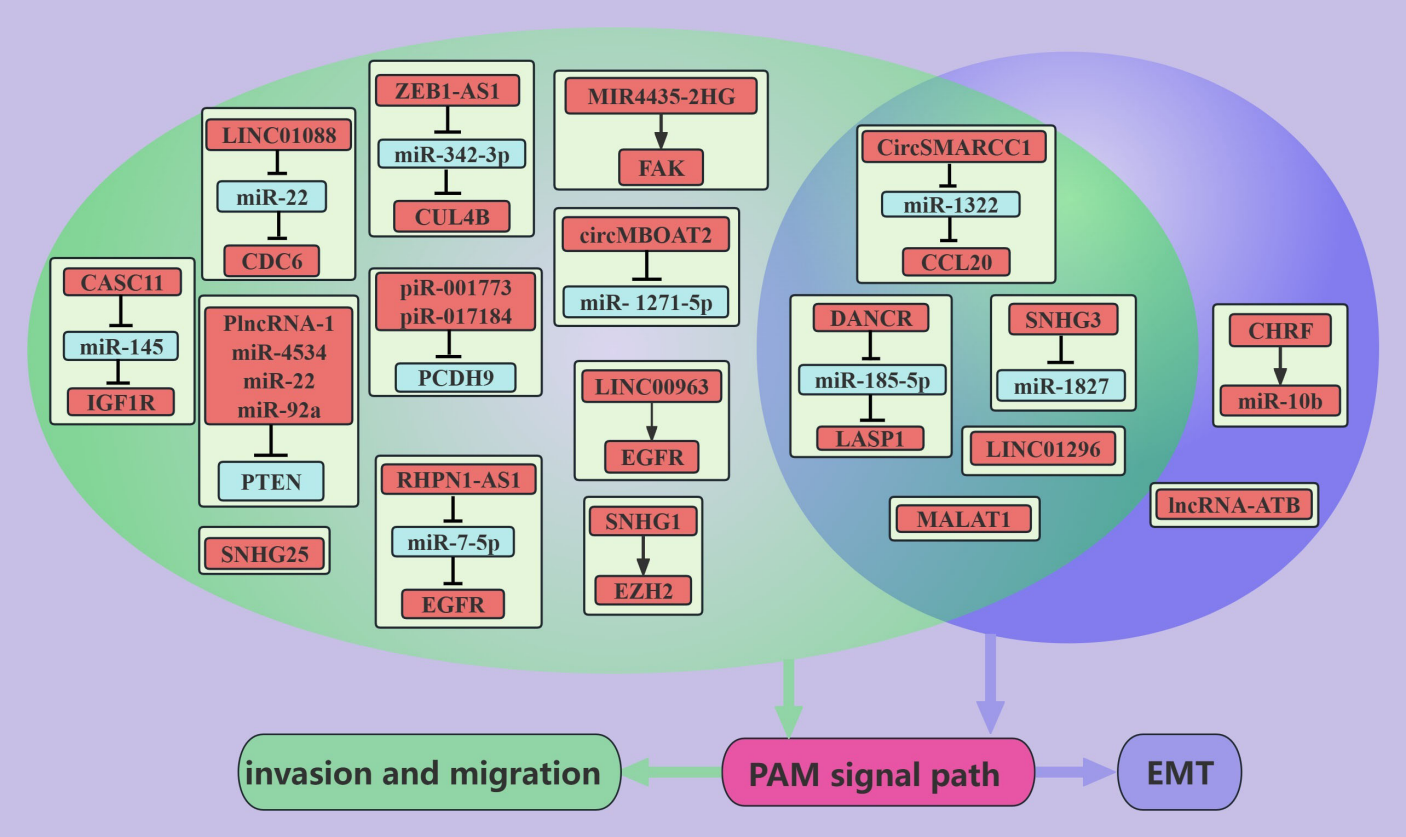
ncRNAs promote PCa metastasis through the PAM signaling pathway. This figure illustrates that ncRNAs associated with the PAM signaling pathway promote PCa metastasis through regulating invasion, migration, and EMT. Blue represents the role of suppressing the PAM signaling pathway. Red represents roles that facilitate the PAM signaling pathway. Cyan color represents association with invasion and migration and purple color represents association with EMT.

It is also very common for ncRNAs to positively regulate the PAM signaling pathway and thus promote PCa metastasis(**Figure 2**). For example, the *DANCR* can activate the PAM signaling pathway through the *miR-185-5p*/LASP1 axis to promote the EMT, migration, and invasion of PCa cells (13). The expression of the *DANCR* and LASP1 was also found to be positively correlated with PCa metastasis, whereas the expression of *miR-185-5p* was negatively correlated with PCa metastasis (13). These findings suggest that the lncRNA *DANCR*/*miR-185-5p*/LASP1 axis plays an important role in the process of PCa metastasis. Dysregulation of EGFR promotes the progression of PCa to bone metastasis (79), and both *LINC00963* and the lncRNA *RHPN1-AS* can contribute to the overexpression of the EGFR gene and increase the migration and invasion of PCa cells; these effects may be mediated via the PAM pathway (12, 23). These findings suggest that drugs that target EGFR potentially inhibit PCa metastasis. PTEN is a tumor suppressor molecule that acts on PIPs. *miR-22, miR-4534, miR-92a*, and *PlncRNA-1* can reduce PTEN expression, thereby promoting the migration and invasion of PCa cells (31, 49, 53, 55). CircRNAs are stably expressed because they exist in a cyclic form. *CircSMARCC1*- and *CircMBOAT2*-overexpressing PC3 cells were injected into the tail vein of nude mice, and more pulmonary and/or abdominal metastases were detected in the nude mice than in the control group (64, 65). Second, *in vitro* experiments have demonstrated that *circSMARCC1* promotes the EMT, migration, and invasion of PCa cells through *miR-22*/CCL20 and that *circMBOAT2* promotes the migration and invasion of PCa cells through *miR-1271-5p*/mTOR (64, 65). These effects may be mediated via the PAM signaling pathway (64, 65). pCDH9, which is a member of the calcineurin superfamily, inhibits the metastasis of hepatocellular carcinoma, gastric carcinoma, and malignant melanoma (80–82). In PCa cells, *piR-001773* and *piR-01718* inhibit PCDH9 and abolish the inhibitory effect of PCDH9 on the PAM signaling pathway, thus promoting the migration and metastasis of PCa cells (67).

Obesity is strongly associated with PCa, with an 8–11% increase in specific mortality in advanced PCa patients who are obese compared with nonobese advanced PCa patients, and the presence of obesity also decreases the efficacy of surgical treatments, radiotherapy and ADT in the treatment of PCa (83). Mediators between obesity and PCa may include insulin and the IGF-axis, sex hormone concentrations, and adipokine signaling (83). PC3 cells with low expression of Apelin, which is an adipokine, were implanted into the prostates of nude mice, and Apelin promoted the liver metastasis and bone metastasis of PC3 cells by increasing the level of TIMP2 (60). Further studies revealed that Apelin increases the level of *miR-106a-5p* through the C-Src/PI3K/AKT axis and that *miR-106a-5p* sponges TIMP2, thereby promoting the migration and invasion of PCa cells (60). IGF1R is a membrane receptor of IGF, and more than 90% of the regions in PCa tissue sections are positive for IGF1R (84). The lncRNA *CASC11* can upregulate the PAM signaling pathway through IGF1R, thereby promoting the migration and invasion of PCa cells (21). These findings suggest that drugs that target IGF1R potentially inhibit PCa metastasis. Therefore, with further in-depth studies of obesity and PCa, obesity has been shown to promote PCa metastasis, and weight loss is likely beneficial for the treatment of PCa.

*MALAT1* mediates the development and metastasis of digestive system and sex hormone-related tumors, and *MALAT1* levels in plasma and urine could be a marker for the diagnosis and prediction of PCa metastasis and recurrence (85). *MALAT1* can promote the EMT, migration, and metastasis of PCa cells by activating the PAM signaling pathway (26, 27), and METTL3 acts as a methyltransferase to methylate *MALAT1* adenosine and thus stabilize its expression (26).

In addition to these novel ncRNAs, *LINC01088*/miR-22 (11), *ZEB1-AS1*/*miR-342-3p *(15), *SNHG3*/*miR-1827 *(18), *SNHG1 *(19), *SNHG25 *(29), *LINC01296 *(32), and *MIR4435-2HG *(34) can promote the migration, invasion or/and EMT of PCa cells via the PAM signaling pathway. Also worth mentioning is *miR-106a-5p*, which is positively regulated by the PAM signaling pathway and promotes PCa migration and invasion (60).

### 3.2 Tumor growth

Tumor growth represents a dysregulation of the balance between cancer cell generation and cell loss; cancer cell generation occurs mainly through cell division, and cancer cell loss occurs mainly through apoptosis and autophagy. In PCa cells, ncRNAs can regulate tumor growth through the PAM signaling pathway, and the elucidation of these mechanisms can provide guidance for the clinical treatment of PCa.

#### 3.2.1 Promotion of tumor growth

**Figure 3:**
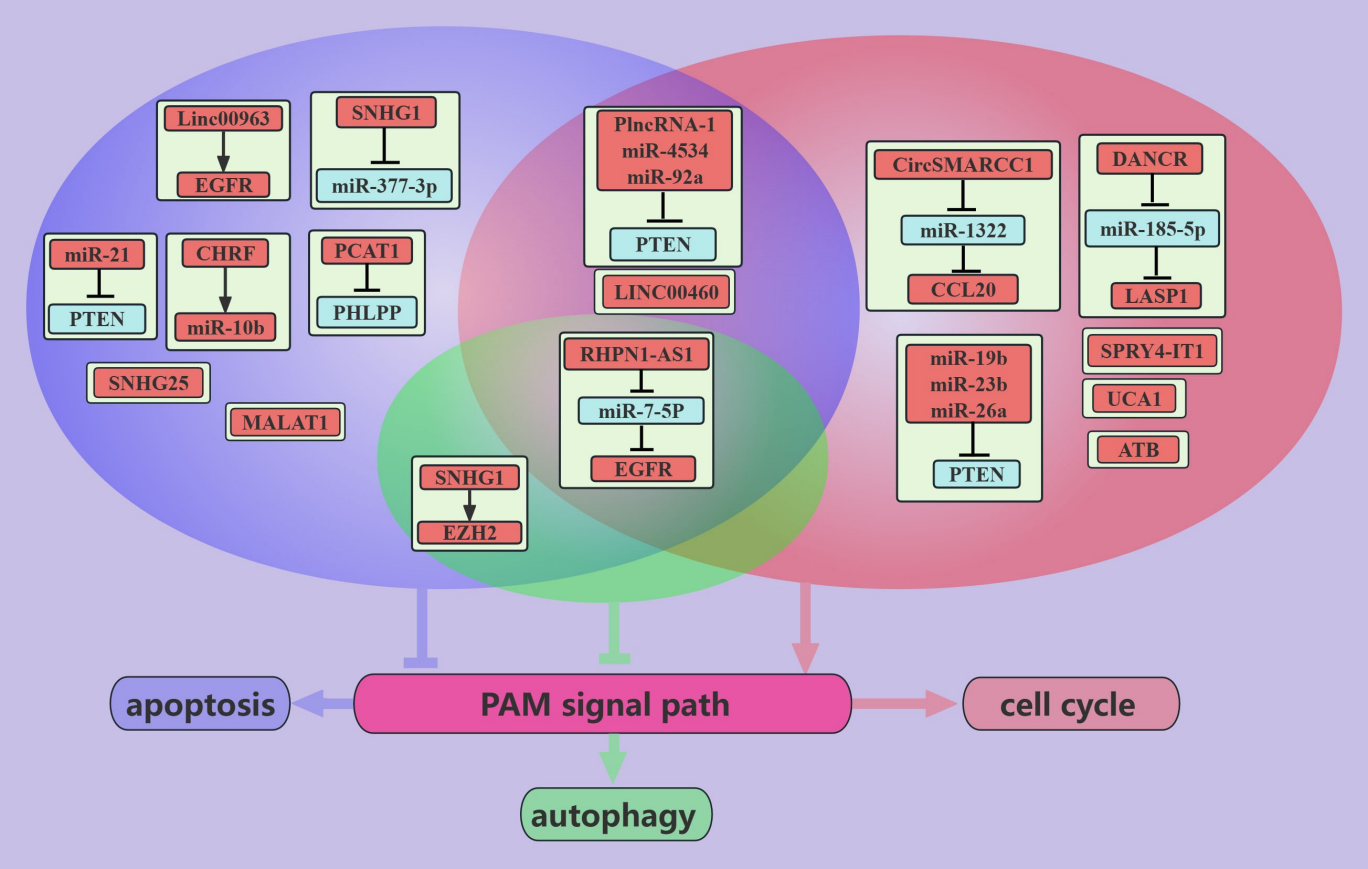
ncRNAs promote PCa growth through the PAM signaling pathway. This figure illustrates that ncRNAs associated with the PAM signaling pathway promote PCa growth through regulating apoptosis, autophagy, and cell cycle. Blue represents the role of suppressing the PAM signaling pathway. Red represents roles that facilitate the PAM signaling pathway. Cyan color represents an association with autophagy, purple color represents an association with apoptosis, and light red color represents an association with the cell cycle.

NcRNAs associated with the PAM signaling pathway promote PCa growth (**Figure 3**). These ncRNAs can promote cancer cell growth via the PTEN-dependent induction of cell cycle progression through cell cycle checkpoints (86). For example, in PCa cells, *miR-4534* promotes the passage of cancer cells through the G0/G1 phase (49); *PlncRNA-1* promotes the passage of cancer cells through the G2/M phase (31); and *miR-19b, miR-23b, miR-26a*, and *miR-92a* promote the passage of cancer cells through the G1/S phase via the cell cycle protein D1 (54). In addition, ncRNAs, such as *miR-4534, PlncRNA-1, miR-92a*, and *miR-21*, can inhibit apoptosis to promote PCa growth via PTEN (31, 49, 50, 55). Interestingly, *miR-21* inhibits apoptosis in chemotherapy-resistant PCa cells, which may be mediated by *miR-21* inhibition of cancer cell exocytosis of toxic drugs taken up via PTEN (50). The hypoxic environment of PCa can induce tumor progression and metastasis (87). PCa cells express high levels of *SPRY4-IT1* in hypoxic environments, and *SPRY4-IT1* may promote the growth of cancer cells by regulating the PAM pathway and cell cycle progression (36). High expression of *UCA1* was observed in radiotherapy-resistant PCa, in contrast to the radiotherapy-sensitive PCa (35). *UCA1* may promote the passage of cancer cells through the G0/G1 phase via the PAM pathway [90]. Moreover, EGFR expression is higher in CRPC than in ADPC (23). Linc00963 was found to transactivate EGFR and mediate the apoptosis of CRPC cells (23). In addition, in PCa, *RHPN1-AS1*/*miR-7-5P*/EGFR could promote cancer cell growth through the PAM signaling pathway (12). This may be mediated by the PAM axis, which promotes apoptosis and autophagy and induces cancer cells to pass the G2/M checkpoint (12).

#### 3.2.2 Inhibition of tumor growth

**Figure 4:**
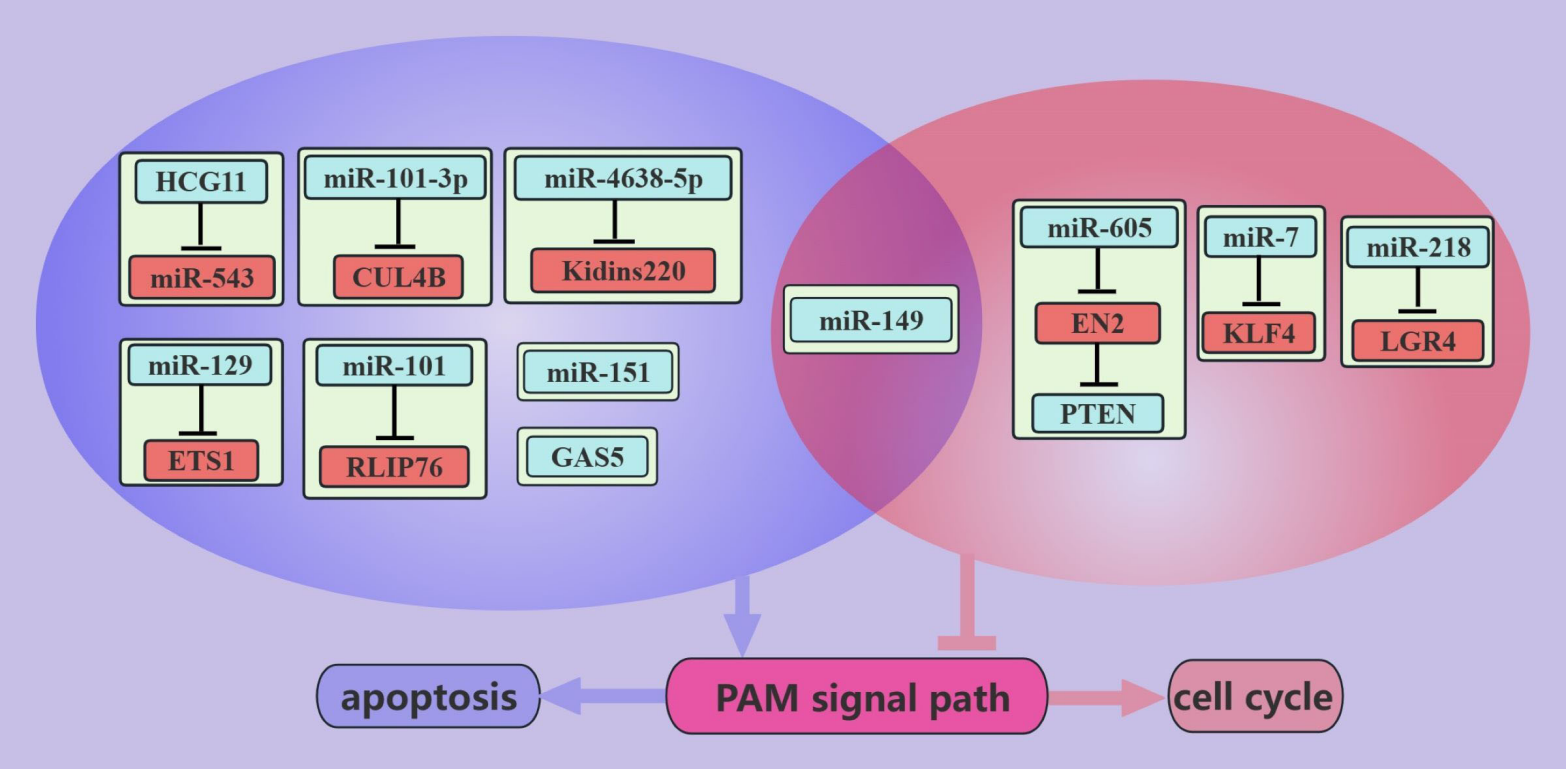
ncRNAs inhibit PCa growth through the PAM signaling pathway. This figure illustrates that ncRNAs associated with the PAM signaling pathway inhibit PCa growth by regulating apoptosis and cell cycle. Blue represents the role of suppressing the PAM signaling pathway. Red represents roles that facilitate the PAM signaling pathway. purple color represents an association with apoptosis and light red color represents an association with the cell cycle.

ncRNAs inhibit PCa growth through the PAM signaling pathway (**Figure 4**). PCa is an androgen-dependent tumor, and *miR-149* can inhibit AR signaling (52). In addition, *miR-149* can target AKT1 to inhibit the proliferation of CRPC cells, which may be mediated by promoting apoptosis and inducing G1/S phase arrest in cancer cells (52). *miR-7* can inhibit the proliferation of PCa cells, possibly because *miR-7* can increase the nuclear localization of P21 through the KLF4/PI3K/AKT axis, which in turn prolongs the cell cycle [91]. Interestingly, *miR-7* increases P-P21 expression but does not seem to interfere with apoptosis (45).

IL-6 stimulation increases PCa viability and invasiveness, suggesting that PCa is closely associated with inflammation (88). *miR-218* inhibits cell cycle progression through LRG4, and this inhibition is suppressed in the presence of IL-6 (48). Therefore, anti-inflammatory therapy can be used as an adjuvant treatment for PCa. RLIP76 depletion therapy can cause the nearly complete regression of PCa cell xenografts and can improve sensitivity to radiotherapy (89). *miR-101* can target and inhibit RLIP76, thereby promoting the apoptosis of PCa cells. *miR-101*-based depletion therapy targeting RLIP76 is promising for the treatment of PCa (42). *miRNA-605* was found to differentiate between inert and aggressive PCa, and further studies found that *miRNA-605* can arrest the cell cycle in the G0/G1 phase by the EN2/PTEN axis (58). In addition, some miRNAs, such as *miR-27a* and *miR-135a*, are negatively regulated by the PAM signaling pathway. These miRNAs arrest PCa cells in the G1/S phase and promote apoptosis through MAP2K4 and RBAK, respectively (62, 63).

### 3.3 Angiogenesis

**Figure 5:**
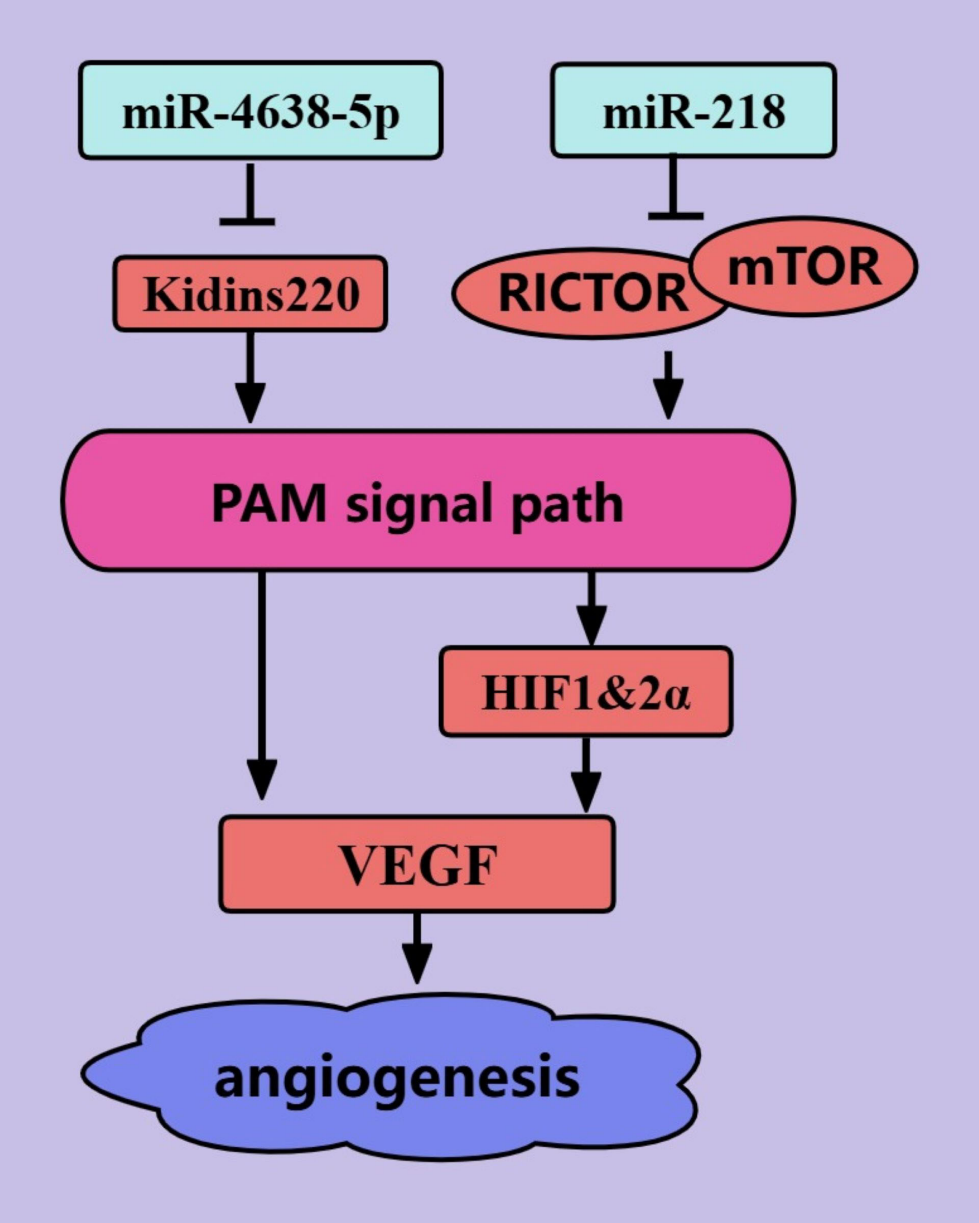
ncRNAs regulate PCa angiogenesis through the PAM signaling pathway. The blue color represents the inhibition of angiogenesis in PCa. The red color represents the promotion of angiogenesis in PCa.

Rapid tumor proliferation is inevitably accompanied by neoangiogenesis because without blood vessels to provide nutrients for tumor cells, the tumor size cannot exceed 1–2 mm (90). NcRNAs regulate PCa angiogenesis through the PAM signaling pathway (**Figure 5**). The interaction of VEGF with VEGFR on the surface of vascular endothelial cells (ECs) is crucial for the proliferation, migration, and tube formation of ECs (91). *miR-4638-5p* can inhibit the secretion of VEGF by PCa cells, thereby inhibiting neovascularization (43). *miR-4638-5p* may inhibit the PAM signaling pathway by targeting the inhibition of Kidins220, thereby reducing the production of VEGF (43). In addition, *miR-218* can inhibit VEGFA secretion by PCa cells, thereby inhibiting neovascularization (46). The effect of *miR-218* on VEGFA is mediated by the RICTOR/AKT/HIF1&2α axis (46). To date, we have elucidated the mechanism by which miRNAs regulate angiogenesis in PCa cells through the PAM signaling pathway.

### 3.4 Tumor stemness

**Figure 6:**
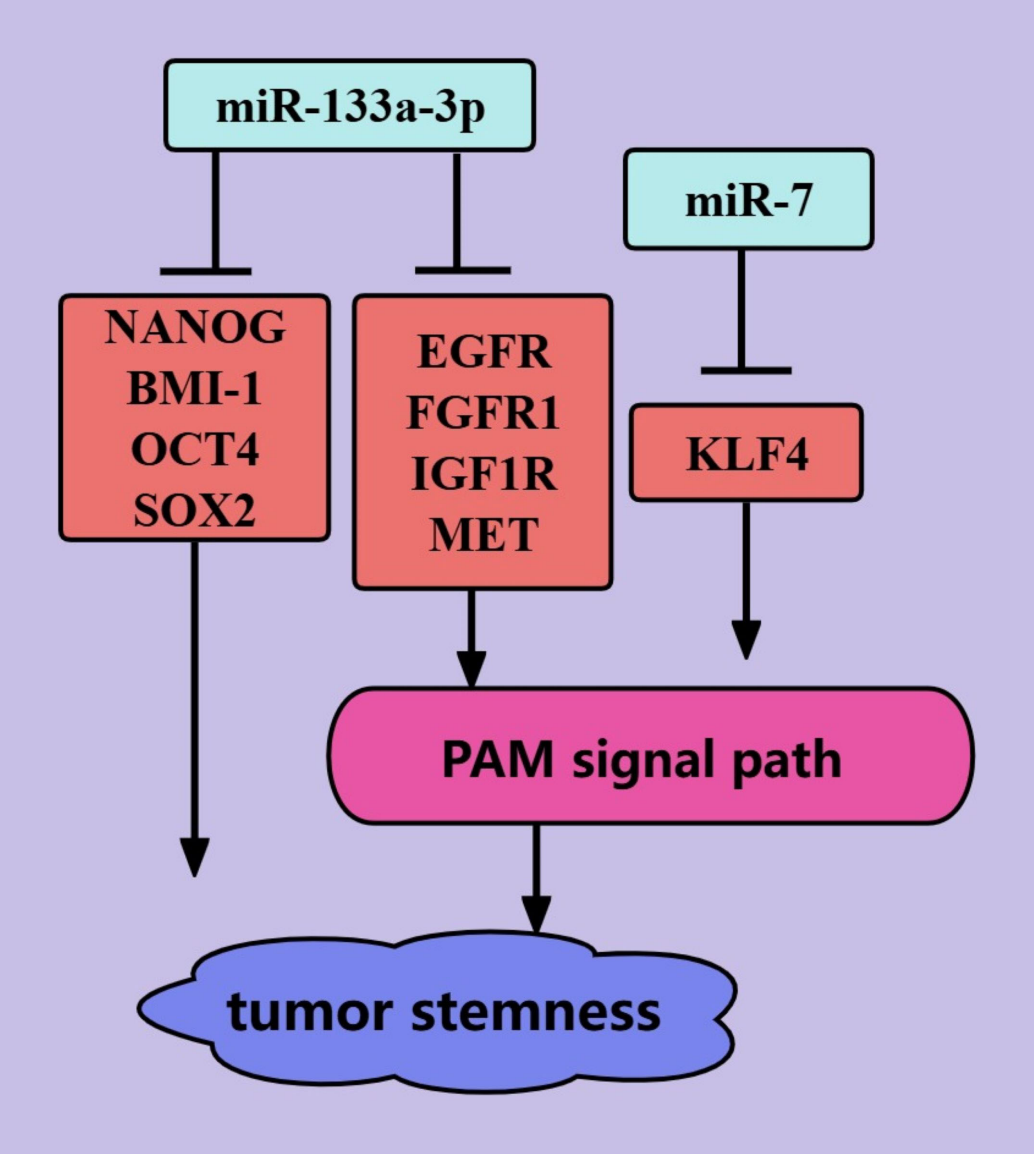
ncRNAs regulate PCa tumor stemness through the PAM signaling pathway. The blue color represents the inhibition of PCa tumor stemness. The red color represents the promotion of PCa tumor stemness.

The presence of cancer stem cells (CSCs) in tumors is an important cause of tumor treatment failure and tumor recurrence, and tumors are considered to be cured only when CSCs are completely eliminated (92). miRNAs mediate the biological regulation of CSCs through the PAM signaling pathway (**Figure 6**) (92). *miR-133a-3p* can inhibit PCa stemness through the PAM signaling pathway. This may be mediated by *miR-133a-3p*, which targets and inhibits EGFR, FGFR1, IGF1R, and MET (44). *miR-133a-3p* was also shown to inhibit the expression of some stemness-related factors, such as NANOG, BMI-1, OCT4, and SOX2 (44). In addition, *microRNA-7* can inhibit PCa stemness by targeting and inhibiting the stemness factor KLF4, possibly because the stemness factor KLF4 activates the transcription of P110δ (the catalytic subunit of PI3K) (45). *MicroRNA-7* inhibits PCa stemness through KLF4 in several generations of cells and prevents the transition from nonstem cells to stem cells (45). Thus, *miR-133a-3p* and *microRNA-7* are promising targets for PCa treatments that target tumor stemness.

## 4 Clinical roles of ncRNAs in the PAM pathway

The ncRNAs associated with the PAM signaling pathway in PCa are of clinical significance (**Table 2**).

**Table 2:** Clinical applications of ncRNAs associated with the PAM signaling pathway in PCa.

	ncRNA	Expression in PCa	Diagnosis	Prognosis	Clinical significance	Ref.
LncRNA	LINC01088	↑	Profitable	Disease-free survival	—	(67)
	RHPN1-AS1	↑	Profitable	—	—	(78)
	DANCR	↑	Profitable	Overall 5-year survival	Pathological grade and tumor metastasis	(34)
	MBNL1-AS1	↓	Profitable	—	—	(31)
	ZEB1-AS1	↑	Profitable	—	—	(77)
	HCG11	↓	Profitable	—	Tumor metastasis	(90)
	GAS5	Associated with SNPs	—	Overall survival	Gleason score and tumor stage	(93)
	SNHG3	↑	Profitable	Overall survival	—	(65)
	SNHG1	↑	Profitable	Overall survival	T stage	(12, 24)
	CASC11	↑	Profitable	—	Gleason score	(26)
	CHRF	—	—	—	—	(52)
	LINC00963	CRPC↑vs. ADPC	Profitable	—	—	(58)
	PCAT1	CRPC↑vs. ADPC	Profitable	Overall survival and recurrence-free survival	—	(84)
	NEAT1	↑	Profitable	—	Tumor metastasis and PSA recurrence	(94)
	MALAT1	↑	Profitable	Tumor recurrence rate	Gleason score, lymphatic node transfer and Quercetin therapy	(41, 76)
	LINC00460	↑	Profitable	—	T stage and Gleason score	(17)
	SNHG25	↑	Profitable(AUC=0.915)	Disease-free survival	—	(47)
	lncRNA-ATB	↑	Profitable(AUC=0.931)	Biochemical recurrence-free survival	Histologic grade, preoperative PSA level, pathologic stage, Gleason score, lymph node metastasis, vascular lymphatic infiltration, and biochemical recurrence	(95)
	PlncRNA-1	↑	Profitable	—	T stage	(96)
	LINC01296	↑	Profitable	Biochemical recurrence-free survival	Preoperative PSA level, lymph node metastasis, Gleason score, and tumor stage	(97)
	AC245100.4	↑	Profitable	—	—	(98)
	MIR4435-2HG	↑	Profitable	—	—	(99)
	UCA1	Radiotherapy-resistant cells ↑ vs. radiotherapy-sensitive cells	Profitable	—	Radiotherapy sensitivity	(54)
	SPRY4-IT1	↑	Profitable	—	Gleason score	(83)
	GDPD4-2	↓	Profitable	—	Astragaloside IV-PESV therapy	(100)
miRNA	miR-139-5p	—	—	—	—	(4)
	miR-151	↓	Profitable	—	Chemosensitivity	(28)
	miR-101-3p	↓	Profitable	—	TNM stage, tumor size, Gleason score, and preoperative PSA level	(51)
	miR-129	↓	Profitable	—	—	(101)
	miR-101	—	—	—	—	(102)
	miR-4638-5p	CRPC↓vs. ADPC	Profitable	—	—	(91)
	miR-133a-3p	↓	Profitable	Bone metastasis-free survival	Gleason score, T stage, N stage, M stage, and bone metastases	(87)
	MicroRNA-7	↓	Profitable	—	—	(27)
	miR-218	↓	Profitable	—	Tumor metastasis	(35, 68, 70)
	miR-4534	↑	Profitable(AUC=0.9)	Overall survival, recurrence-free survival	Gleason score, T stage, PSA recurrence, and genistein treatment	(63)
	miR-21	Chemoresistant cells ↑ vs. chemosensitive cells	Profitable	—	chemotherapy resistance	(103)
	miR-182	↑	Profitable	—	Distant metastasis, lymph node metastasis, and tumor size	(6)
	MicroRNA-149	—	—	—	—	(104)
	miR-125b	↓	Profitable	—	—	(11)
	miR-22	↑	Profitable	—	—	(11)
	miR-19b	—	—	—	—	(45)
	miR-23b	—	—	—	—	(45)
	miR-26a	—	—	—	—	(45)
	miR-92a	—	—	—	—	(45, 105)
	miR-151a-3p	↓	Profitable	—	—	(101)
	miR-188-5p	—	—	Biochemical recurrence-free survival and overall survival	Lymph node metastasis, T stage, preoperative PSA level, vascular lymphatic infiltration, biochemical recurrence, and chemosensitivity	(106)
	miR-605	↓	Profitable	—	—	(107)
	miR-137	—	—	—	—	(64)
	miR-106a-5p	↑	Profitable	—	—	(74)
	miR-106b	—	—	—	Prolactin therapy	(13)
	miR-27a	↓	Profitable	—	—	(89)
	miR-135a	↓	Profitable	Biochemical recurrence-free survival	Gleason score, T stage, biochemical recurrence	(42)
circRNA	CircSMARCC1	↑	Profitable(AUC=0.713)	—	Gleason score, and T stage	(108)
	circMBOAT2	↑	Profitable	Disease-free survival	Gleason score and T stage	(21)
	cir-ITCH	↓	Profitable	—	—	(71)
piRNA	piR-001773	↑	Profitable	—	Gleason score and T stage	(109)
	piR-017184	↑	Profitable	—	Gleason score	(109)

↑ represents elevated levels of ncRNA in cancerous tissues compared to non-cancerous tissues. ↓ represents that ncRNA is reduced in cancerous tissues compared to non-cancerous tissues. ↓vs. represents a lower content of the former than of the latter. ↑vs. represents a higher content of the former than the latter.

### 4.1 Diagnosis

The diagnosis of PCa is the basis of treatment, and ncRNAs that are involved in the PAM pathway were found to help confirm the diagnosis of PCa by ROC curve analysis. For example, an analysis of 52 paired samples revealed an AUC of 0.915 for *SNHG25 (* 29), an analysis of 57 paired patients revealed an AUC of 0.931 for *LncRNA-ATB (* 30), and a study of 28 unpaired patients reported an AUC of 0.9 for *miR-4534 (* 49). We aimed to detect and diagnose prostate cancer by noninvasive methods, and we detected elevated *circSMARCC1* levels in the plasma of PCa patients compared with those in the plasma of noncancerous patients (64). ROC curve analysis revealed that the AUC of *CircSMARCC1* was 0.713, which was not as good as that of PSA in identifying PCa (64). However, the specificity of *circSMARCC1* was high enough to discriminate between patients with PCa and those with BPH, and *CircSMARCC1* levels were also found to be elevated only in colorectal cancer tissues (97). ncRNAs that are associated with the PAM signaling pathway can also predict various stages of PCa. For example, lower expression of *miR-185-5p*, *miR-218*, and *miR-133-3p* and higher expression of *DANCR* were observed in metastatic patients than in nonmetastatic PCa patients (13, 44, 46). Interestingly, *miR-185-5p* expression was more significantly decreased in patients with bone metastatic PCa than in patients without bone metastatic PCa (44). High expression of *PCAT1* and *NEAT1* suggests CRPC (24, 25), high expression of *miR-21* suggests chemoresistance (50), and high expression of *UCA1* suggests radioresistance (35). Thus, ncRNAs are expected to be novel markers for the diagnosis of PCa.

### 4.2 Prognosis

Prognostic information on PCa helps in the selection of optimal treatment strategies (108), and the levels of ncRNAs that are related to the PAM signaling pathway can indicate the prognosis of PCa patients. These ncRNAs are closely associated with overall survival, progression-free survival, biochemical recurrence-free survival, bone metastasis-free survival, and recurrence-free survival in PCa patients. For example, the levels of *lncRNA-ATB*, *LINC01296*, and *miR-188-5p* can be used as independent prognostic factors for the biochemical recurrence-free survival of PCa patients (30, 32, 57). The *miR-133a-3p* level can be used as an independent prognostic factor for the bone metastasis-free survival of PCa patients (44). Second, these ncRNAs are closely associated with pathological features that affect prognosis; for example, plasma *CircSMARCC1* levels are positively correlated with the Gleason score and T stage in patients with PCa (64). In addition, single nucleotide polymorphisms (SNPs) of ncRNAs have the potential to predict the prognosis of PCa (17). For example, two SNPs on chromosome *GAS5* are correlated with overall survival, Gleason score, and TNM stage in patients with PCa (17).

### 4.3 Treatment

**Figure 7:**
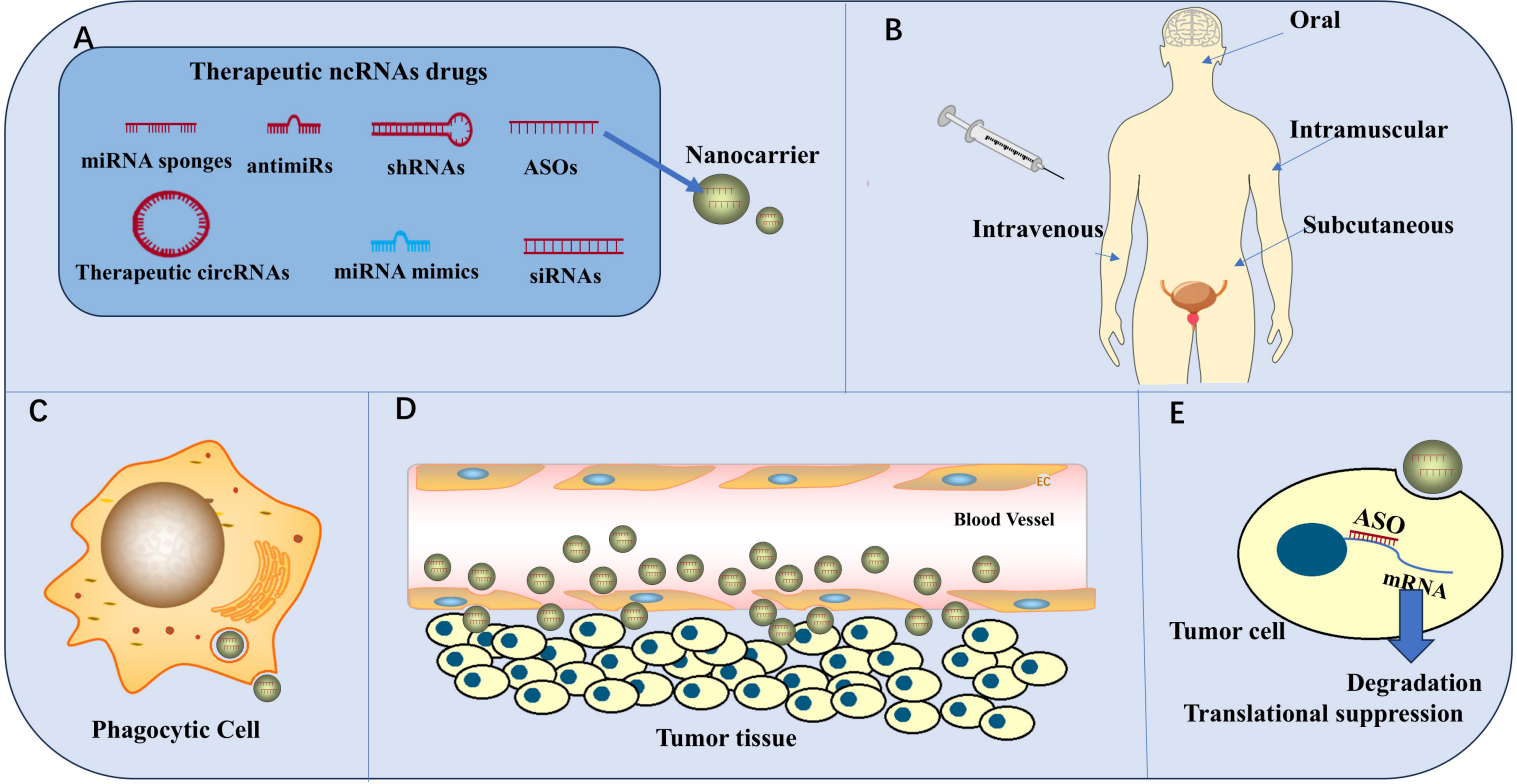
ncRNAs for clinical therapy. **(A)** Therapeutic ncRNA drugs include ASOs, siRNAs, shRNAs, RNA sponges, antmiRs, therapeutic circRNAs, and miRNA mimics. ASOs were the first ncRNA drugs to be developed. Nanomaterials are the most common carriers for ncRNA drugs. **(B)** The administration routes of ncRNA drugs include oral, intramuscular, subcutaneous, and intravenous administration **(C)** Phagocytic cells destroy the efficacy of the drug. **(D)** Drugs cross blood vessel walls through intercellular spaces or channels and endocytosis. **(E)** ASOs enter tumor cells to cause mRNA degradation and translation suppression, thus achieving anti-tumor effects.

The targeted regulation of ncRNAs can inhibit PCa growth, metastasis, angiogenesis, and drug resistance via the PAM signaling pathway; this provides a basis for developing PCa therapies that target ncRNAs. For example, agomir-133a-3p inhibits bone metastasis of PCa in mice (44), knockdown of *UCA1* reduces radiotherapy resistance in PCa cells (35), and inhibition of *miR-21* increases the chemosensitivity of PCa cells (50). ncRNAs are natural molecules in the body, and they can interfere in a given pathway with broad specificity via multiple targets or target different pathways to inhibit tumors (99), leading to increased benefits in ncRNA therapy. For example, *miR-218* can target LGR4 to reduce AKT levels in PCa cells, target RICTOR to inhibit the phosphorylation of AKT at Ser473, and target GAB2 to disrupt the coexistence environment of PI3K and PIPs to inhibit the PAM signaling pathway (46–48). In addition, *miR-218* not only inhibits the PAM pathway but also interferes with the Wnt/β-catenin pathway in PCa cells (48). Drugs that specifically target ncRNA therapeutics include antisense oligonucleotides(ASOs), siRNAs, shRNAs, RNA sponges, antmiRs, therapeutic circRNAs, miRNA mimics (**Figure 7A**), and the drugs that are currently being developed are mainly miRNA-based siRNAs and antisense oligonucleotides (99). However, therapies that target ncRNAs have limitations associated with specificity, delivery, and tolerance when translated into the clinic, resulting in the failure of the development of these drugs (99). The use of novel drug delivery strategies, such as the possible use of nanomaterials, liposomes, cationic polymers, etc., for drug delivery, has shown promising advantages in overcoming these difficulties (94). Nanocarrier-based drug therapies for ASOs are currently the most hotly researched option (**Figure 7**), and better structures have been achieved in other diseases.

To date, there are only two ncRNA drugs in human trials regarding PCa. Apatorsen (OGX-427) is a 2′-methoxyethyl-modified ASO that inhibits Hsp27 expression. A Phase I clinical trial including 42 (22 CRPC patients) showed that OGX-427 was tolerated at the highest dose (101). Then a phase II trial showed that OGX-427+prednisone did not improve the 12-week progression-free period in CRPC patients compared to prednisone alone, but only significantly reduced PSA in patients, so further clinical trials of OGX-427 are in limbo (95) There is another drug Custirsen (OGX-011) that is an ASO targeting clusterin inhibition. A meta-analysis including 3 RCTs pointed out that OGX-011 did not significantly improve OS in mCRPC patients, so it has been terminated from development in the clinic (102).

A cohort study including 1132 patients revealed that the use of traditional Chinese medicine reduced PCa-related mortality (32.8% vs. 21.9%) compared with the nonuse of traditional Chinese medicine (105). Traditional Chinese medicine (TCM) has multitarget properties, and TCM is involved in the entire process of PCa treatment (100). Recently, traditional Chinese medicine has been shown to modulate ncRNAs and thus treat PCa; for example, quercetin can downregulate *MALAT1* in a time- and dose-dependent manner to inhibit the growth, migration, invasion, and EMT of PCa cells. This may be mediated by targeting the PAM signaling pathway via *MALAT1 *(27). Astragaloside IV-PESV can upregulate *GDPD4-2* to inhibit the PAM pathway and PCa growth (37). However, silencing *GDPD4-2* does not completely block the antitumor effect of astragaloside IV-PESV, suggesting that astragaloside IV-PESV may also act on other targets to inhibit PCa growth (37). In addition, traditional Chinese medicine can be used together with other antitumor drugs not only to exert antitumor effects alone but also to produce synergistic effects and improve the sensitivity of tumor cells to antitumor drugs (100, 106). For example, the use of quercetin together with docetaxel can significantly improve the antitumor effect of docetaxel on chemotherapy-resistant PCa (106). Moreover, traditional Chinese medicine makes it easier to achieve clinical translation than specific drugs do; thus, traditional Chinese medicine has broad prospects in the treatment of tumors.

## 5 Discussion

This paper summarizes the types, functions and specific mechanisms of action of ncRNAs that are associated with the PAM signaling pathway in PCa cells. Most of these ncRNAs act at the transcription level. lncRNAs and circRNAs mostly function as miRNA sponges, and miRNAs and piRNAs mostly function by suppressing mRNA transcription. In addition, some ncRNAs, mainly lncRNAs, act posttranscriptionally to regulate the PAM pathway. The PAM signaling pathway is abnormally activated in PCa cells and interacts with the RAS/MAPK, AR, and WNT pathways (110). This paper explore the roles of ncRNAs in the apoptosis, cellular autophagy, cell cycle progression, EMT, invasion, migration, angiogenesis, and tumor stemness of PCa cells via the PAM pathway. We analyzed the effects of these ncRNAs on PCa cell apoptosis, autophagy and cell cycle regulation in PCa growth and their effects on EMT, invasion and migration in PCa metastasis, and we presented these functions in the form of Venn diagrams, which can allow a more comprehensive understanding of the regulatory effects of these ncRNAs on PCa growth and metastasis. We found that some ncRNAs affect PCa growth and metastasis through dual or triple mechanisms, which more clearly highlights the importance of certain ncRNAs and may provide guidance for subsequent studies. For example, *miR-149* can inhibit the growth of PCa cells by affecting apoptosis and cell cycle progression, and it can inhibit PCa metastasis through EMT, migration, and invasion (52). If the role of *miR-149* is further explored, it can be found that *miR-149* can inhibit AR signaling via the PAM pathway (52). In fact, cell growth, metastasis, angiogenesis and tumor stemness are intertwined with each other, and the roles of these ncRNAs in the interactions of these processes can be further explored. In addition, we explored how these ncRNAs affect PCa growth, metastasis, angiogenesis and tumor stemness via the PAM pathway. We found that the regulatory effect of these ncRNAs on PCa angiogenesis depended on VEGF; that EGFR mediated the effects of *miR-7-5p*, *miR-133a-3p*, and *LINC0096* on PCa growth, metastasis, and tumor stemness; and that PTEN mediated the effects of *miR-181a-5p, miR-4534, miR-22, miR-21, miR-26a, miR-23b, miR-92a*, and *miR-19* on PCa growth and metastasis.

We also discussed the prospects for the clinical application of these ncRNAs in diagnosis, prognosis, and treatment. In terms of diagnosis and prognosis, most of the ncRNA content in cancer tissues can be detected, and some of the ncRNA content shows high sensitivity for diagnosing PCa and determining patient prognosis. However, these ncRNAs are not easy to sample and analyze, they cannot be included in a dynamic detection index, and most ncRNAs do not have high specificity for the diagnosis of PCa. It is hoped that more *circSMARCC1*-like ncRNAs will be discovered and that diagnostic and prognostic markers with high specificity will be available in the bloodstream. Therapeutically, drugs that target these ncRNAs are based mainly on ASO, siRNAs, shRNAs, RNA sponges, antmiRs, therapeutic circRNAs, and miRNA mimics, and these drugs have shown excellent therapeutic effects in animal and cellular experiments. However, when these drugs are used in humans, their efficacy is greatly reduced. For example, Custirsen is an antisense nucleotide drug that targets human CLU mRNA and was developed to treat chemotherapy-resistant mCRPC patients (98). However, in a phase III clinical trial that included 635 patients, it was shown that there was no statistically significant difference in overall survival in the Custirsen + chemotherapy group compared to the chemotherapy control group (14.2 moon vs. 13.4 moon, p = 0.529) (98).

This loss of efficacy is mainly because the drug will face delivery challenges. ncRNA drugs cannot be delivered efficiently to the cells of interest due to their own instability and lack of cell specificity. Secondly, crossing cell membranes is also a great challenge. Scientists have proposed to solve the delivery problem by altering the internal chemical modifications, such as 2′-deoxy-2′-fluoro-RNA, 2′-O-methyl-RNA, the phosphorothioates, and bicyclic nucleic acids, of ncRNA drugs to improve resistance to nucleases and enhance interaction with proteins to improve stability and uptake (109). Custirsen was dual-modified with alternating 2′-O-methyl and the phosphorothioates, but phase III clinical trials showed that the effect was not significant and was halted (98, 111). Apatorsen (OGX-427) is also an ASO doped with phosphorothioates and 2′-methoxyethyl modification to prolong half-life and improve specificity aimed at treating mCRPC patients (101). However, phase II trials have shown that Apatorsen (OGX-427) does not alter mCRPC disease progression, but only reduces PSA concentrations in patients (95). These failures suggest the need to improve the efficacy of the drug in other ways, so recently scientists have thought of solving this problem through novel delivery systems. For example, nanoparticles, which can be synthesized from liposomes, polymers, micelles, proteins, antibodies, gold nanoparticles, USPIO nanoparticles, and nanotubes, in addition to enhancing the stability of RNA drugs, due to their small size can also enhance the permeability and retention (EPR effect) of tumor tissue to increase the concentration of the drug inside the tumor tissue (112). The miR-145 therapy based on gold nanoparticle-based nanocarrier and miR-34a therapy based on micelle-based nanocarrier have achieved good therapeutic efficacy in cellular and animal tests (103, 107). Based on nanoparticles, some scientists combine Ultrasound-induced microbubble cavitation technology to increase the permeability of cell membranes and the capillary gap, which further facilitates the entry of drugs into cancer cells (93). However, these have yet to be proven in human trials and the safety of nanoparticles needs to be explored further. The side effects of nanoparticles are also due to the EPR effect, which leads to the aggregation of nanoparticles in vascular leakage tissues. The side effects are caused by reactive oxygen species (ROS), DNA damage, modification of protein structures and functions, and disruption of membrane integrity mechanisms leading to damage in these tissues (113). Viral-constituted delivery carriers have also been believed to enhance the efficacy of RNA drugs by loading the target RNA into the viral genome. Viral-constituted delivery carriers deliver RNA drugs into tumor tissues through viral oncolytic properties and stability. For example, the current delivery of miRNAs for PCa therapy using herpes simplex virus-1 and recombinant adeno-associated virus has shown good results in animal studies (96, 114). However, the exact efficacy has to be observed in humans and the safety is worth considering. In addition to viral delivery and nano-delivery systems, membrane vesicles, bacteria, and ligand-receptor delivery systems are also explored in ncRNA therapy for PCa (99).

In the future, the internal modification of ncRNA itself to make ncRNA more stable, the carrier delivery system to further stabilize the ncRNA and increase the drug concentration in the tumor region, and the ligand-receptor refinement to navigate the drug into the target tumor cells will make ncRNAs as clinical drugs a reality. While these enhance the efficacy of ncRNA drugs, the carrier system itself can cause some toxic effects, which need to be addressed. In addition, the human immune system recognizes single- or double-stranded RNA inside and outside the cell through the PAMP receptor, which reduces the drug’s efficacy and causes an immune-inflammatory response (99). As PCa is a “cold tumor” in immunotherapy, could the immune-inflammatory response by such ncRNAs potentiate the immunotherapy of PCa? Nanocarriers have been shown to enhance the antitumor efficacy and specificity of immunopharmaceuticals through enhanced immunostimulatory activity and favorable modulatory pharmacological properties (104). Therefore, whether treatment with ncRNAs in combination with vectors could make PCa a “hot tumor” in terms of immunotherapy. Currently, the translation from cell and animal trials to clinical trials is the biggest obstacle to ncRNA drug therapy, which may be solved in the future by the combined delivery of ncRNA drugs through multiple mechanisms.

Previously OGX-427 and OGX-011 were considered to be promising for clinical use, but the therapeutic effect was not shown to be significant in clinical trials. Therefore, attempts can be made to develop other ncRNAs for the treatment of PCa. miR-218 gene is located on chromosome 4p15.31, which is transcribed as an intron into an ncRNA and is expressed in various malignant tumors at lower levels than in the surrounding normal tissues (115). It can be used as a tumor suppressor gene. Earlier Katia R M Leite and other scholars have found that miR-218 is highly expressed in metastatic PCa compared to high-grade PCa patients (116). In recent years, many studies have shown that miR-218 may regulate the PAM signaling pathway in PCa, and basically, it works by binding to the 3’-UTR of the downstream genes’ mRNA and blocking the mRNAs’ translation process. miR-218 can target LGR4 to reduce the content of AKT, target RICTOR to inhibit the phosphorylation of AKT ser-473 and target GAB2 to destroy the coexistence environment of PI3K and PIPs to inhibit the PAM signaling pathway (46–48). miR-218 can effectively inhibit the viability, migration, and invasion of PCa cells and inhibit tumor angiogenesis through multi-targeting inhibiting PAM signaling. miR-218’s multi-targeting properties may improve its efficacy in the clinical translational process, and have the opportunity to be applied in the clinic. In addition, miR-133a-3p may play an important role in the treatment of PCa bone metastasis (44). It was shown that upregulation of miR-133a-3p inhibited PCa bone metastasis and stem cell characteristics and that miR-133a-3p levels were strongly associated with bone metastasis-free survival in PCa patients (44). Moreover, miR-133a-3p can target multiple receptors, such as EGFR, FGFR1, IGF1R, and MET receptors, to inhibit tumor cells (44). Therefore, the development of miR-133a-3p as a novel drug for PCa patients with bone metastasis is promising, but the specific effects are still waiting to be observed in clinical trials.

There is a lack of effective treatment for mCRPC. From the current clinical data, the PAM signaling pathway inhibitor (Ipatasertib) brings the best efficacy, which can prolong the survival of patients by 2 months. However, there is still much room for improvement (117). Based on the above, this review summarizes the ncRNAs in the PAM signaling pathway of PCa and tries to find AKT inhibitor breakthroughs from the function and clinical use of these ncRNAs for better treatment of PCa. We also discussed the prospect of clinical translation of ncRNAs in PCa, but we found that only a few clinical studies show that ncRNA drugs’ efficacy in PCa is still poor and that ncRNAs are unstable. There is still a long way to go before they can be applied to the clinic; In addition to the ncRNA strategy, there may be other pathways implicit in the PAM signaling pathway to treat PCa, which is the shortcoming of this review. Therefore, we wrote this review for the next study on the use of ncRNA in PCa.

We can refer to ncRNA drug development strategies that have been successfully used in the clinic in other diseases, and more data are needed in the future to explore the next step of ncRNA therapies for PCa. This review investigates ncRNAs under the PAM pathway in PCa to provide a reference for the next step in the study of ncRNAs for new drugs or new strategies.
